# Association between Neuron-Specific Enolase, Memory Function, and Postoperative Delirium after Transfemoral Aortic Valve Replacement

**DOI:** 10.3390/jcdd10110441

**Published:** 2023-10-25

**Authors:** Jonathan Nübel, Charlotte Buhre, Meike Hoffmeister, Stefanie Oess, Oliver Labrenz, Kerstin Jost, Michael Hauptmann, Julika Schön, Georg Fritz, Christian Butter, Anja Haase-Fielitz

**Affiliations:** 1 Department of Cardiology, University Hospital Heart Centre Brandenburg, Brandenburg Medical School Theodor Fontane, 16321 Bernau, Germany; jonathan.nuebel@immanuelalbertinen.de (J.N.); charlotte.buhre@mhb-fontane.de (C.B.); christian.butter@immanuelalbertinen.de (C.B.); 2 Faculty of Health Sciences (FGW), Joint Faculty of the University of Potsdam, The Brandenburg Medical School Theodor Fontane and the Brandenburg Technical University Cottbus-Senftenberg, 16816 Cottbus, Germany; meike.hoffmeister@mhb-fontane.de (M.H.); stefanie.oess@mhb-fontane.de (S.O.); michael.hauptmannn@mhb-fontane.de (M.H.); 3 Institute of Biochemistry, Brandenburg Medical School Theodor Fontane, 14770 Brandenburg an der Havel, Germany; 4 Department of Psychology, Brandenburg Medical School Theodor Fontane, 16816 Neuruppin, Germany; oliver.labrenz@mhb-fontane.de (O.L.); kerstin.jost@mhb-fontane.de (K.J.); 5 Institute of Biostatistics and Registry Research, Brandenburg Medical School Theodor Fontane, 16816 Neuruppin, Germany; 6 Anesthesia and Intensive Care, University Hospital Ruppin Brandenburg (UKRB), Brandenburg Medical School Theodor Fontane, 16816 Neuruppin, Germany; j.schoen@ukrb.de; 7 Department of Anesthesiology, Intensive Care and Pain Therapy, University Hospital Heart Centre Brandenburg, Brandenburg Medical School Theodor Fontane, 16321 Bernau, Germany; georg.fritz@immanuelalbertinen.de; 8 Institute of Social Medicine and Health System Research, Otto von Guericke University Magdeburg, 39120 Magdeburg, Germany

**Keywords:** TAVR, postoperative delirium, memory function, cognitive assessment, neuron-specific enolase

## Abstract

Introduction: Although transfemoral aortic valve replacement (TAVR) is a safe treatment for elderly patients with severe aortic valve stenosis, postoperative microembolism has been described. In this secondary endpoint analysis of the POST-TAVR trial, we aimed to investigate whether changes in neuron-specific enolase (NSE)—a biomarker of neuronal damage—are associated with changes in memory function or postoperative delirium (POD). Materials and Methods: This was a prospective single-center study enrolling patients undergoing elective TAVR. Serum NSE was measured before and 24 h after TAVR. POD was diagnosed using CAM-ICU testing. Memory function was assessed before TAVR and before hospital discharge using the “Consortium to Establish a Registry for Alzheimer’s Disease” (CERAD) word list and the digit span task (DST) implemented in “∆elta-App”. Results: Subjects’ median age was 82 years (25th to 75th percentile: 77.5–85.0), 42.6% of subjects were women. CERAD scores significantly increased from pre- to post-TAVR, with *p* < 0.001. POD occurred in 4.4% (6/135) of subjects at median 2 days after TAVR. After TAVR, NSE increased from a median of 1.85 ng/mL (1.30–2.53) to 2.37 ng/mL (1.69–3.07), *p* < 0.001. The median increase in NSE was 40.4% (13.1–138.0) in patients with POD versus 17.3% (3.3–43.4) in those without POD (*p* = 0.17). Conclusions: Memory function improved after TAVR, likely due to learning effects, with no association to change in NSE. Patients with POD appear to have significantly higher postoperative levels of NSE compared to patients without POD after TAVR. This finding suggests that neuronal damage, as indicated by NSE elevation, may not significantly impair assessed memory function after TAVR.

## 1. Introduction

Transcatheter aortic valve replacement (TAVR) is a minimally invasive procedure for patients with severe and symptomatic aortic stenosis (AS). Even though it has become the standard treatment in elderly patients, TAVR is not without risks. Patients undergoing this procedure may experience severe hemodynamic instability, microembolism, or stroke with a risk of cerebral ischemia [1,2]. Although the incidence of clinically silent peri-interventional cerebral embolic lesions after TAVR is high, persistent neurological impairment in elderly patients seems to be low [3]. Postoperative delirium (POD) occurs in about 10–20% of patients undergoing TAVR and is associated with an increased risk of mortality [4,5]. Even in patients who recovered from POD, it can lead to long-term brain atrophy and cognitive impairment [6].

As a common biomarker of cerebral damage, neuron-specific enolase (NSE) has shown to be elevated in patients who underwent TAVR, potentially indicating neuronal damage during the procedure [7]. NSE is a cell-specific isoenzyme of the glycolytic enzyme enolase (Enzyme Commission (EC) classification number 4.2.1.11); it is mainly expressed in the cytoplasm of neurons and neuroendocrine cells [8]. When axons are damaged, NSE is upregulated to maintain homeostasis, making it a marker of ischemic brain damage [9]. In clinical practice NSE is frequently used for predicting neurological outcome after cardiac arrest with good prognostic accuracy [10]. Postoperative serum concentrations of NSE and corresponding kinetics after cardiac surgery have a high predictive value to early neuropsychological and neurobehavioral outcome [11]. Also, NSE predicted adverse neurologic outcomes and extent of stroke after cardiac surgery [12]. A recent meta-analysis underlines that high postoperative NSE may predict postoperative cognitive dysfunction [13].

As a partial aspect of neurological function, memory function has a crucial impact on patients’ independence and quality of life [14,15]. Because elaborated neurocognitive testing is challenging in elderly patients undergoing TAVR, the impact of well-described microembolization on POD development or cognitive function after TAVR still remains unclear. A simple test to detect changes in memory function and screen for patients at risk for POD using neuro-biomarkers would be desirable for daily patient care. This pilot study aims to explore the association between NSE as a biomarker of neurological damage and POD development as well as app-based cognitive testing after TAVR.

## 2. Material and Methods

### 2.1. Patients

This prospective cohort study enrolled adult patients undergoing elective TAVR at the University Hospital Heart Center Brandenburg between October 2020 and March 2022. Inclusion criteria comprised patients with severe symptomatic aortic stenosis who were scheduled for TAVR and were classified as high surgical risk patients according to the European Society of Cardiology guidelines [16]. Exclusion criteria were emergency surgery, chronic dialysis, and lack of written informed consent for study participation. For this exploratory secondary endpoint analysis of the POST-TAVR trial (DRKS00020813), only patients who had a complete preoperative and postoperative memory test as well as pre- and postoperative serum samples of NSE were included.

**Figure 1:**
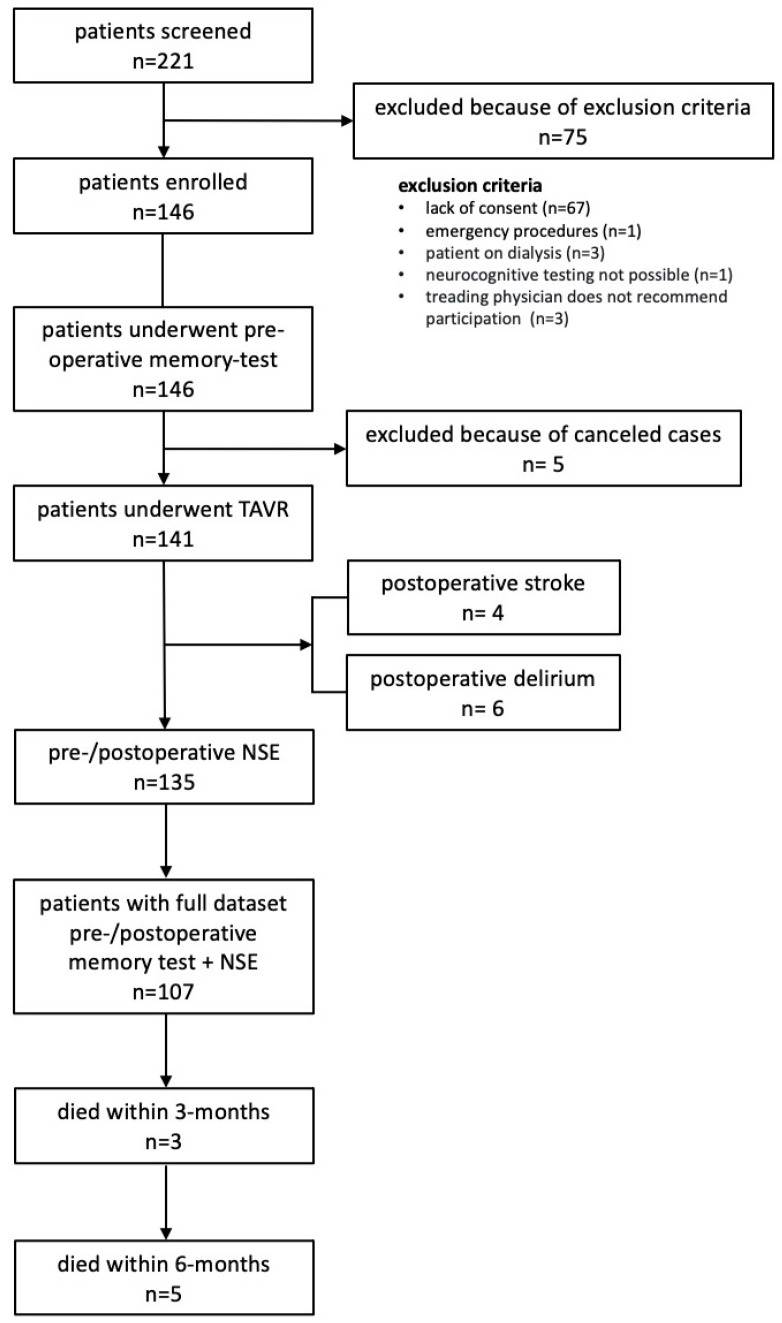
Patient flow through the study.

For all patients, a multidisciplinary valve team, including interventional cardiologists, cardiothoracic surgeons, and cardiovascular anesthesiologists, was involved in assigning surgical or nonsurgical treatment. Patients and general practitioners were followed up 3 months after discharge. Patient flow is shown in Figure 1. The study was approved by a local ethics committee (E-01-20191006).

### 2.2. TAVR Procedure

The majority of patients were admitted on the day before the procedure. The TAVR device was delivered through femoral approach in all patients. TAVR was performed under fluoroscopy guidance with the use of contrast media. Procedures were performed under local anesthesia with conscious sedation or general anesthesia with endotracheal intubation. The prosthesis size was determined using preprocedural multi-slice computed tomography angiogram findings.

### 2.3. Neurocognitive Function Assessment

Preoperative and postoperative cognitive function was assessed with validated tests (CERAD word-list learning and recall and Digit Span Task, DST) using the “∆elta-App”(version number 1.2.1). Patients with POD were tested after full recovery from POD. Full recovery from POD was defined as obtaining a negative result on the CAM-ICU before further postoperative cognitive assessment. ∆elta is a digital application and a speech recognition technology that uses artificial intelligence for automated speech analysis. The certified medical product understands human speech, recognizes semantic relationships, and draws conclusions from the information collected. All test results underwent manual cross-checking by a trained psychology student. A third-generation iPad Air from Apple with the operating system iPadOS 14.4 was used to conduct the test.

The CERAD Word List (WL) is a memory assessment tool that measures immediate (WL learning) and delayed (WL recall) recall for new and non-associated verbal information.

In WL learning assessment, 10 unrelated words were presented acoustically, and the patients had to repeat each of them aloud directly after presentation. After the list was completed, patients were asked to immediately recall as many words as possible, regardless of order. This procedure was repeated twice with words presented in different order. The WL learning (or immediate recall) score is the sum of the numbers of correctly recalled words in each of the three trials (i.e., maximum of 30 words). In addition, delayed recall (WL recall) was performed at the end of the cognitive test battery: patients were asked to recall as many of the previously presented words without further exposure (maximum of 10 words).

The Digit Span Test (DST forward) is a neuropsychological assessment tool that measures auditory attention and short-term memory capacity [17]. A series of digits is presented, and the participant’s task is to immediately repeat the sequence of digits in the correct order. The length of the digit sequences gradually increases by 1 digit (starting with 3 up to a maximum of 9 digits) if the participant successfully recalls at least one of the sequences of a particular length. If the participant makes an error, the test usually stops, and the longest correctly recalled sequence is taken as the participants’ digit span.

### 2.4. Screening for Postoperative Delirium (POD)

Patients were screened for POD during their intermediate care or intensive care unit (ICU) stay, or in case of any neurocognitive abnormalities, with the “Confusion Assessment Method for the Intensive Care Unit” (CAM-ICU). The CAM-ICU assesses 4 features: (1) the acute onset of mental status changes, or a fluctuating course; (2) inattention; (3) altered levels of consciousness; and (4) disorganized thinking. It is positive if a patient manifests feature 1 and 2, plus either feature 3 or 4 [18]. To avoid missing POD outside the screening interval, we included physician discharge diagnoses of delirium.

### 2.5. Neuron-Specific Enolase (NSE) Measurement

Blood samples for NSE biomarker measurement were obtained right before TAVR and at 24 h postoperatively. Until the end of the study period, samples for biomarker analysis were stored at −80 °C and dispatched on dry ice for analysis. Coinvestigators performing biomarker assays were blinded to patient details and memory function classification. All measurements were performed in technical duplicates. NSE serum levels were measured using commercially available immunoassay Human Enolase 2/Neuron-specific Enolase (DY5169, RnD Systems, Minneapolis, MN, USA) according to the manufacturer’s instructions. For better comparability with previously published literature, the results of the assay were converted from pg/mL to ng/mL. Optical densities were determined using a microplate reader (TriStar^2^ S LB 942, Berthold Technologies, Bad Wildbad, Germany). Calculation of results were performed by generation of the corresponding standard curves and sample concentrations were determined using 4-parameter logistic (4PL) curve fitting (MyAssays online data analysis tool, www.myassays.com, accessed on 20 September 2023). Laboratory investigators were blinded to the sample sources and clinical outcomes.

### 2.6. Data Collection

Medical records were reviewed until hospital discharge. The following information was obtained: demographics, comorbidities, procedural characteristics including valve type and size, intraprocedural and postprocedural complications during the index hospital stay (cardiac decompensation, need for packed red blood cells, sepsis, or septic shock), laboratory parameters, length of stay in hospital after TAVR, in-hospital mortality, as well as discharge status. Rehospitalization within 90 days after discharge, 90-day mortality, and major adverse cardiac events (MACE) were assessed by a questionnaire sent to the treating primary care physician. In addition, patients were interviewed 90 days after hospital discharge using a structured telephone interview.

### 2.7. Statistical Analysis

Variables are described using medians and 25th to 75th percentiles or means and standard deviations for memory function (±SD) for continuous measures, as appropriate, and proportions for categorical measures. Comparisons between groups were performed using chi-square or Fisher’s exact test for categorical variables, and Student’s *t*-test or the Mann–Whitney *U* test for continuous variables, as appropriate. Correlations between variables were reported using 2-sided Spearman test.

To describe the preoperative cognitive status of the patients, test scores of the CERAD word-list tasks were transformed into age-, sex- and education-referenced standardized scores (z-values) as implemented in ∆elta-App. For further group analyses, participants with z-values ≤ −1.28 (10% percentile) were characterized as cognitively impaired (according to the recommendations in the CERAD manual and Lezak et al., 2012) [19].

As practice effects are common in repeated assessments of word-list tests within short test–retest intervals, we paid special attention to patients who did not demonstrate any performance enhancement. We considered the absence of performance improvement or learning effects as diagnostically significant for cognitive impairment. Consequently, we conducted a subgroup analysis and categorized CERAD score changes as either deterioration in memory functions or non-deterioration. We evaluated the association between dichotomized NSE changes (≥20% increase versus <20%) [20] and CERAD deterioration versus non-deterioration. Boxplots show the median, interquartile range, and outliers (as open circles) in the concentration (ng/mL) of NSE.

We performed a linear regression analysis for the endpoint post-memory function adjusted for delta-NSE and pre-memory function.

A *p*-value of less than 0.05 was considered statistically significant. SPSS 29 (IBM, Armonk, NY, USA) was used.

## 3. Results

In total, 146 patients were enrolled, of whom 141 underwent TAVR and 135 had complete measurement of NSE. Patient flow is shown in Figure 1. The median time between preoperative cognitive assessment and TAVR was 1 (1–2) days; between TAVR and postoperative assessment, it was 4 (3–5) days.

### 3.1. Patient Characteristics and Outcome

Overall, median age was 82 years (77.5–85.0) with a Euro-Score II of 8.0% (5.1–13.9). The incidence of POD was 4.3% (6/141) and occurred, in median, on the second postoperative day.

Baseline characteristics, overall and by POD incidence, are shown in Table 1. Patients with and without POD were comparable regarding age, gender, EuroScore II, NYHA class, and most of the comorbidities. A higher proportion of patients with POD presented with hyperlipidemia (five of six, 83.3%) and previous PTCA (five of six, 83.3%) compared to patients with no POD (58/135, 43.0% and 48/136, 35.6%, *p* = 0.09 and *p* = 0.028, respectively). Also, patients with POD had higher NT-proBNP and lower estimated glomerular filtration rate before TAVR compared to patients without POD (Table 1). A balloon-expanding valve was used in 66.6% (four of six) of patients with POD versus 18.7% (25/134) of patients without POD, *p* = 0.02. A postoperative stroke was diagnosed in 2.8% of patients (4/141), and 14.9% (21/141) required a pacemaker implantation after TAVR (Table 2). The median length of stay in hospital was 10 days (8–13), with differences observed in patients with POD (15 days (13–26)) compared to those without POD (10 days (8–13), *p* = 0.003). Overall, 22.3% of patients were rehospitalized within 90 days: 66.7% (two of three) with POD versus 21.0% (21/100) without POD during their index hospital stay (*p* = 0.12). The odds ratio (OR) of hospital readmission within 90 days was 7.52 [95% CI: 0.65–87.0] for patients with POD versus others. Overall, three patients died within 90 days (all without previous POD).

### 3.2. Neuron-Specific Enolase (NSE)

**Figure 2:**
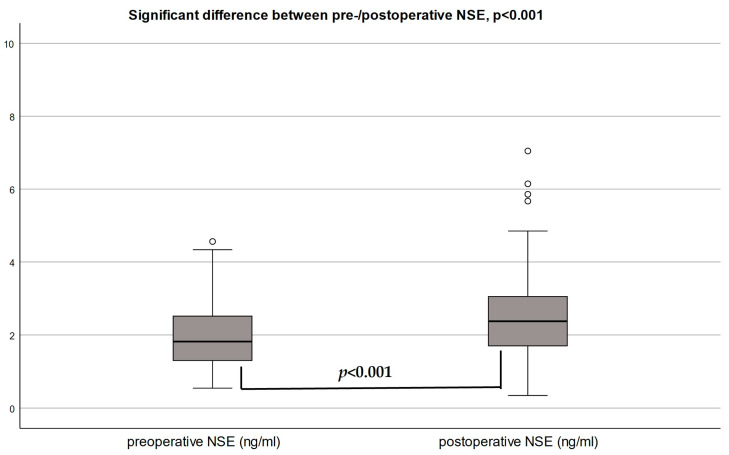

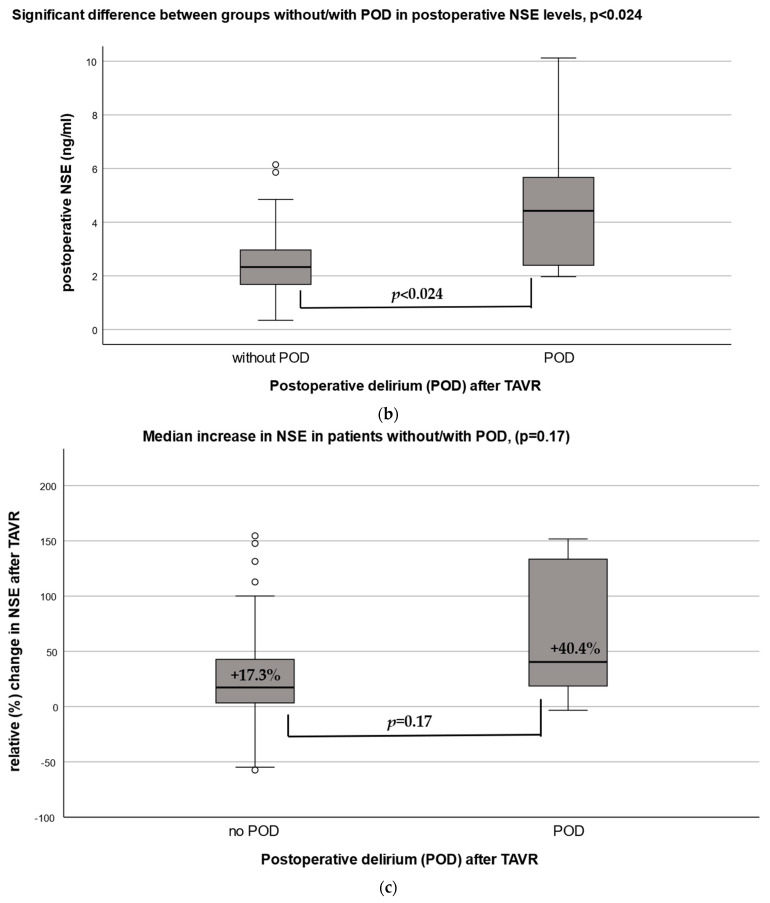
(**a**) Neuron-specific enolase (NSE) before and after TAVR. Comparison of preoperative versus postoperative median serum NSE ng/(mL) concentration. (**b**) Postoperative median concentrations of neuron-specific enolase (NSE) by POD incidence. (**c**) Median increase in % change in NSE by POD incidence. Relative (%) change in NSE levels by POD groups with (+40.3%) and without (+17.3%) postoperative delirium (POD), *p* = 0.17.

The median baseline NSE in patients undergoing TAVR was 1.85 ng/mL (1.30–2.53), and the median postoperative NSE was 2.37 ng/mL (1.69–3.07) 24 h after TAVR. The highest postoperative NSE level was 19.01 ng/mL. NSE levels increased by 41.9%, on average, after TAVR, with a significant median increase of 0.31 ng/mL (0.05–0.86), *p* < 0.001 (Figure 2a).

There was no difference in median postoperative NSE levels between patients undergoing TAVR with conscious sedation (2.52 ng/mL, 1.79–2.96) or general anesthesia (2.29 ng/mL, range: 1.65–3.11), *p* = 0.19.

In total, 45.9% of the patients (62/135) had an increase in NSE of more than 20% from baseline. Compared to the group of patients with an NSE elevation < 20%, patients with an NSE elevation > 20% had an OR 2.45 [95% CI 0.43–13.84], *p* = 0.31, for POD. We identified interoperative switch to heart surgery (*p* < 0.001) and intraprocedural hemodynamic relevant pericardial effusion (*p* < 0.001), as well as preoperative hyperlipoproteinemia (*p* = 0.044) and preoperative percutaneous transluminal coronary angioplasty (*p* = 0.037), as independent risk factors for POD. None of these correlated with delta-NSE (interoperative switch to heart surgery (*p* = 0.095)), intraprocedural hemodynamic relevant pericardial effusion (*p* = 0.97), preoperative hyperlipoproteinemia (*p* = 0.36), and preoperative percutaneous transluminal coronary angioplasty (*p* = 0.42). Also, delta-NSE did not correlate with volume of contrast media used during the implantation (r = −0.086, *p* = 0.33) or with procedural time (r = 0.05, *p* = 0.58). There was no significant correlation between patients with postoperative stroke (*n* = 4) and postoperative NSE levels (*p* = 0.33).

### 3.3. Post-NSE in Patients with and without POD

After TAVR, patients with POD had a higher median NSE level compared to patients without POD after TAVR (4.42 ng/mL vs. 2.33 ng/mL, *p* = 0.024) (Figure 2b). The median relative increase in NSE was 40.4% (13.1–138.0) in patients with POD versus 17.3% (3.3–43.4) in those without POD (*p* = 0.17) (Figure 2c). Adjusted for preoperative NSE levels, the relative increase in NSE had an OR 1.0 [95% CI 0.995–1.01], *p* = 0.40, for POD.

### 3.4. Preoperative and Postoperative Memory Function

Word-list scores significantly increased from pre- to post-TAVR test sessions, with *p* < 0.001 for both WL learning and WL recall. The mean digit span before TAVR was 5.39 numbers, with no significant changes after TAVR (*p* = 0.5). Participants with and without POD did not differ in change of memory functions for WL learning (*p* = 0.66), WL recall (*p* = 0.25), and DST (*p* = 0.40).

### 3.5. Neuron-Specific Enolase (NSE) and Memory Function

Complete data sets with pre- and post-TAVR assessment were available for WL learning in *n* = 108 patients, for WL recall in *n* = 107 patients, and for DST in *n* = 109 patients. There was no significant correlation between the change in NSE and the change in WL learning (r = −0.11, *p* = 0.27), WL recall (r = −0.16, *p* = 0.10), and DST (r = −0.07, *p* = 0.47). In a linear regression adjusted for preoperative memory function, delta-NSE had no significant effect on post-memory function for WL learning (B = −0.21, *p* = 0.31), WL recall (B = −0.09, *p* = 0.46), and DST (B = −0.04, *p* = 0.51).

Preoperative median NSE level amounted to 1.94 ng/mL (1.33–2.65) in the group with impaired WL learning (82/146) versus 1.75 ng/mL (1.25–2.35) in the group with preserved function (64/146). This difference was not significant, *p* = 0.61. Also, for WL recall, preoperative median NSE levels of 1.83 ng/mL (1.24–2.61) and 1.90 ng/mL (1.4–2.37) for the groups of impaired (109/145) and unimpaired patients (36/145), respectively, did not differ (*p* = 0.36).

Pre-interventional memory function did not correlate with pre-interventional NSE in WL learning (r = −0.12, *p* = 0.16) and WL recall (r = −0.01, *p* = 0.96).

### 3.6. Binary Memory Performance in CERAD and Neuron-Specific Enolase

We dichotomized memory performance changes from pre- to post-TAVR into deterioration vs. no deterioration (% with deterioration in WL learning: 24.1, 27/112; % with deterioration in WL recall: 14.4, 16/111). There were no significant differences in the NSE change by binary memory performance for WL learning (with deterioration: 0.31 ng/mL [0.14–0.71] vs. without deterioration: 0.30 ng/mL [0.02–0.93], *p* = 0.59) and WL recall (0.37 ng/mL [0.13–0.85] vs. 0.29 ng/mL [0.03–0.82], *p* = 0.40). There was no difference in the occurrence of deteriorations in WL learning (*p* = 0.92) and WL recall (*p* = 0.59) between the group with > 20% postoperative NSE increase compared to the group without.

## 4. Discussion

To the authors’ knowledge, this is the first prospective pilot cohort study of TAVR patients to investigate the association of POD and memory function with NSE, as an established biomarker of neuronal injury. Using app-based, standardized cognitive tests in patients undergoing TAVR, we analyzed the association between serum NSE levels, development of POD, and postoperative memory function. The incidence of POD after TAVR was 4.4%. Overall, concentrations of serum NSE increased after TAVR. Postoperative concentrations of NSE were higher in patients with POD compared to those without POD. In 45.9% of patients, NSE increased by more than 20%. However, there was no correlation between the change in NSE and memory function.

Several studies have examined the association between TAVR and POD. In an updated systematic review and meta-analysis, Ma X. et al. included 413,389 patients and calculated a pooled mean incidence of POD of 9.8% (95% CI: 8.7–11.0%) [5]. In our study, the number of patients with POD after TAVR was small. The results of this pilot study may lack transferability and may be susceptible to detection and selection bias. Nonetheless, NSE, a biomarker of cerebral damage, was able to detect a biologically plausible signal and distinguished patients with and without POD after TAVR. These findings should be discussed under the aspects of possible detection and selection bias. POD can be difficult to diagnose: it can present subtly, in many different ways and with rapid changes, and can be mistaken for other conditions [21]. The American College of Critical Care Medicine recommends the use of CAM-ICU [22] as an instrument for POD screening; nevertheless, its sensitivity is not perfect [23]. In our study, patients were only screened for POD during their IMC/ICU stay or in case of any neurocognitive abnormalities during hospitalization. This may have resulted in missed diagnoses of delirium, especially in its rapid changing manifestations or sub-manifestations. To avoid missing POD outside the screening, we likewise included physician discharge diagnoses of delirium.

According to the investigators, some patients with risk factors for POD, such as preoperative impaired cognitive function, advanced dementia, or high frailty and elderliness [24], declined to participate due to the fear of “failing” or “performing poorly” in the cognitive test. A selection bias must therefore be assumed. On the other hand, it can be noted that our institution has well-structured TAVR procedures that can reduce the probability of delirium. These include effective analgesia, intraoperative cerebral monitoring, the use of dexmedetomidine, and a high level of expertise of the nursing staff to accompany, manage, and reorient postoperative patients [25].

In line with previous published data, we found that 45.9% of participants had an increase of more than 20% from baseline NSE after TAVR, which is similar to the 47.5% reported by Ghanem et al. [20]. Gailiušas et al. previously showed that NSE was significantly increased in patients with POD compared to patients without POD (*p* = 0.042) after coronary artery bypass grafting [26]. Mietani et al. demonstrated that postoperative elevated NSE in cancer surgery discriminated, with significantly high accuracy, between patients with and without clinically diagnosed POD (AUC of 0.87 [95% CI 0.79–0.95], *p* < 0.0001) [27]. Although, in our pilot study, NSE was also increased after TAVR and had a low but significant correlation to POD, the predictive power of NSE for POD could not be confirmed in our study for TAVR patients. This may be attributed to the low number of POD diagnoses and the fact that none of our study patients reached the cut-off value of 201.2 ng/mL used by Mietani et al. [27].

Prior to TAVR implantation, our cohort had, on average, relatively low word-list learning and recall scores (see the z-scores in Table 3), suggesting severe preprocedural impairment in memory function (at least when procedural differences with the original CERAD version and deficient matching with the available normative data are neglected). After TAVR, WL learning and WL recall scores increased and showed the previously reported [28], and thus expected, improvements. Thus, despite preprocedural impairments and TAVR implantation, our patient group did show, on average, practice/learning effects. We, therefore, have no evidence for a systematic negative effect of TAVR on memory function as measured by WL learning and recall and digit span task. This finding is consistent with previously published data in this field that could not show a general impact of TAVR on cognitive functions [29,30]. Of course, the size of the expected practice/learning effects in our study is difficult to estimate. Thus, some of our participants may even experience improved memory functions after TAVR. At the same time, *n* = 38 participants of our study cohort showed deterioration in at least one of the memory tasks. Despite this individual variation, change in memory function (t1–t0 difference in memory scores) did, however, not correlate with pre-/postoperative or delta-NSE.

In the context of previously published MRI-based studies, NSE increase appears to be further indication that microemboli may occur regularly after TAVR [3]. However, at least in our study, it did not lead to any significant impairment in memory function after TAVR. Talbot-Hamon et al., however, pointed out that “*by using mean scores, the larger pool of individuals with cognitive stability or improvement is likely to dilute and mask the small but clinically relevant subset of individuals with cognitive decline after TAVR*” [31]. Given the potential underdiagnosis and under-reporting of adverse cognitive outcomes, we attempted to identify the subgroup of patients with poor postoperative memory performance. However, even within this subgroup of patients with worse memory function, we did not find a correlation with NSE.

There are also some limitations to our study. Unlike the original version of CERAD word-list tasks, in which words are presented visually, we here used auditory presentation of the word list in the “∆elta-App”. This may have contributed to the relatively low pre-TAVR test scores. However, there is also evidence that memory for word lists is not affected by administration modality [32]. Heterogeneity in clinical testing and lack of standardization also limit the comparability of published work in this area. The central limitation of our study, however, was its small size. A relatively low incidence of POD led to only six cases, which limits the strengths of our findings and renders them mainly exploratory.

NSE has not only its value in cognitive function, but it also has a high diagnostic efficacy when screening for small cell lung cancer (SCLC), even if its predictive and prognostic role remains controversial [33]. The best diagnostic performance for SCLC was obtained at an NSE cut-off of 25 ng/mL, which was not reached by any individual in our study population. In addition, all patients received a contrast-enhanced CT of the chest to the groin for preoperative TAVR-planning and vascular and aortic anulus imaging, which often reveals an incidental malignant finding [34]. Superficial findings consistent with SCLC would have been identified previously in the population.

Establishing NSE as a biomarker for cognitive outcomes after any medical intervention encounters, to this day, several problems: differences between laboratories, the lack of diagnostic thresholds, the optimal diagnostic timing for sampling NSE, and the diagnostic significance of NSE fluctuations between measurements. One difficulty we encountered throughout the study was the varying threshold limits for NSE described in the literature [35]. Zandenbergen et al. describe a hard cut-off value of >33 ng/mL for a poor outcome after CPR [36]. In the critical discussion of this difficulty, Chung-Esaki et al. concluded that the laboratory measurements interfere with the absolute value of NSE and, rather, the change in percentual value determines the cognitive outcome. This group suggests a measurement of NSE after 24, 48, and 72 h after cardiac arrest to be a prognostic biomarker for cognitive outcome [35]. To what extent this can be transferred to NSE measurements after TAVR needs to be evaluated. To conclude, NSE is not yet a routine biomarker with set cut-off values.

Postoperative memory decline is challenging to predict, even if it has a high value for patient quality of life. The difficulty to assess it in daily clinical practice suggests that it may currently be underdiagnosed. Objective and simple-to-use measures are desired, especially for elderly and frail patients who may not be able to undergo extensive and complex psychological testing but require complex interventions like TAVR. Future research should, therefore, examine additional neuro-biomarkers for their predictive value regarding explicit neurological functions, such as memory function, to engage in this tightrope walk.

## 5. Conclusions

We found a relatively low incidence of POD (4.3%), which may have been influenced by diagnostic challenges and patient selection bias. Performance in memory tasks was found to improve after TAVR, likely due to learning effects. No evidence emerged indicating a negative impact on memory function associated with TAVR or indicating elevated postoperative NSE levels. These findings imply that microemboli, as indicated by NSE elevation, may occur after TAVR but may not lead to significant memory impairment. However, additional research in larger patient series is necessary to elucidate NSE’s role as a biomarker in TAVR outcomes due to its complexity and the absence of standardized cutoff values.

## Figures and Tables

**Table 1:** Patient characteristics.

	With POD (*n* = 6)	Without POD (*n* = 135)	Overall (*n* = 141)	*p*-Value
**Demographics**				
Age, years	79 (72.0–84.0)	82 (78.0–85.0)	82 (77.5–85.0)	0.33
Female	3 (50%)	57 (42.2%)	60 (42.6%)	0.70
Body mass index, kg/m^2^	29.0 (24.3–34.4)	27.0 (24.1–31.0)	27.0 (24.2–31.0)	0.43
Smoker	0 (0%)	16 (11.9%)	16 (12.8%)	0.49
EuroScore II (%)	6.5 (4.0–15.1)	8.0 (5.2–4.0)	8.0 (5.1–13.9)	0.50
Education in years	8.0 (8.0–13.5)	10.0 (8.0–14.0)	10 (8.0–13.5)	0.42
**Comorbities**				
Arterial hypertension	5 (83.3%)	109 (80.5%)	114 (80.9%)	>0.99
Coronary heart disease	6 (100%)	85 (63.0%)	91 (64.5%)	0.09
Atrial fibrillation	2 (33.3%)	46 (34.1%)	48 (34.0%)	>0.99
Peripheral arterial occlusive disease	0 (0%)	11 (8.1%)	11 (7.8%)	>0.99
Previous myocardial infarction (6 months)	1 (16.7%)	12 (8.9%)	13 (9.2%)	0.45
Stroke in history	0 (0%)	9 (6.7%)	9 (6.4%)	>0.99
Acute decompensated heart failure	0 (0%)	5 (3.7%)	5 (3.5%)	>0.99
Chronic kidney disease	4 (66.7%)	50 (37.0%)	54 (38.3%)	0.21
Hypertensive heart disease	1 (16.7%)	17 (12.6%)	18 (12.8%)	0.57
Hyperlipoproteinemia	5 (83.3%)	58 (43.0%)	63 (44.7%)	0.09
Diabetes type II (Insulin)	3 (50%)	30 (22.2%)	33 (23.4%)	0.14
Asthma	0 (0%)	6 (4.4%)	6 (4.3%)	>0.99
COPD	0 (0%)	14 (10.4%)	14 (9.9%)	>0.99
Previous PTCA	5 (83.3%)	48 (35.6%)	53 (37.6%)	0.03 *
Previous coronary artery bypass grafting	1 (16.7%)	13 (9.6%)	14 (9.9%)	0.47
Cardiac device	2 (33.3%)	14 (10.4%)	16 (11.3%)	0.14
**New York Heart Association Classification (NYHA) (*n* = 132)**				
NYHA-Classification I	0 (0%)	2 (1.6%)	2 (1.5%)	0.38
NYHA-Classification II	0 (0%)	28 (22.2%)	28 (21%)	
NYHA-Classification III	5 (83.3%)	90 (71.4%)	95 (71.3%)	
NYHA-Classification IV	1 (16.7%)	6 (4.8%)	7 (5.3%)	
**Laboratory parameters before TAVR**				
Serum creatinine, µmol/L	139.0 (108.3–199.3)	87.0 (72.0–114.5)	90.0 (73.0–116.0)	0.003 *
eGFR, mL/min	38.5 (23.0–46.8)	63.0 (47.0–81.5)	62.0 (46.0–80.0)	0.003 *
NT-proBNP, pg/mL	11,486 (2161–22,900)	1343 (714–2959)	1385 (751–3502)	0.06
Hemoglobin, mmol/L	8.2 (7.1–8.8)	8.1 (7.2–8.6)	8.1 (7.2–8.6)	0.77
Serum NSE, ng/mL	2.50 (1.95–4.33)	1.78 (1.25–2.44)	1.85 (1.30–2.53)	0.04 *
**Echocardiographic parameters before TAVR**				
LVEF, %	40 (35–40)	45 (40–60)	55 (40–60)	0.15
TAPSE, mm	18 (17–21)	21 (17–24)	21 (17–24)	0.36

PTCA, percutaneous transluminal coronary angioplasty; CABG, coronary arterial bypass grafting; TAPSE, tricuspid annular plane systolic excursion; LVEF, left ventricle ejection fraction. Self-expanding valve: Evolut™, ALLEGRA™, ACURATE neo™, Portico™. Balloon-expanding valve: SAPIEN™. NYHA missing values: 3/135; * significant *p*-value.

**Table 2:** Procedural characteristics, patient outcome and follow-up.

	With POD (*n* = 6)	Without POD (*n* = 135)	Overall (*n* = 141)	*p*-Value
**Procedural Characteristics**				
Days to TAVI	3 (2–7)	4 (2–6)	4 (2–6)	0.82
Valve type (*n* = 136)				
Evolut R Evolut Pro Sapien 3 **	2 (33.3%) 0 (0%) 4 (66.6%)	81 (62.3%) 25 (19.2%) 24 (18.5%)	83 (58.9%) 25 (17.7%) 28 (19.9%)	0.03 *
Balloon-expanding valve	4/6 (66.6%)	25/134 (18.7%)	29 (20.7%)	0.02 *
Self-expanding valve	2/2 (33.3%)	109/134 (81.3%)	111 (79.3%)	0.02 *
Predilatation	4/6 (66.6%)	92/134 (68.7%)	96 (68.6%)	>0.99
Postdilation	0/6 (0%)	29/134 (21.6%)	29 (20.7%)	0.35
Valve-in-valve	0/6 (0%)	6/134 (4.5%)	6 (4.3%)	>0.99
General anesthesia	6/6 (100%)	104/134 (77.6%)	110 (78.6%)	0.34
Sedation	0/6 (0%)	30/134 (22.4)	30 (21.4%)	0.34
Procedural time, min.	47.0 (40.5–70.0)	51.0 (43.0–62.3)	51.0 (43.0–62.8)	0.68
Volume of contrast media, mL	120 (97–134)	113 (87–148)	113 (87–148)	0.77
Amount of rapid ventricular pacings (RVPs)	3 (2–5)	3 (2–4)	3 (2–4)	0.56
Cumulative RVP time, sec.	52 (39–102)	49.5 (32–67)	50 (32–67)	0.51
**Laboratory markers after TAVR**				
Change in serum NSE, ng/mL **	1.4 (0.26–3.44)	0.3 (0.05–0.81)	0.3 (0.05–0.86)	0.065
Serum creatinine, µmol/L **	131.0 (114.0–323.5)	79.0 (62.0–96.8)	80.0 (62.0–98.0)	<0.001 *
eGFR, mL/min ***	46.5 (33.0–55.5)	73.0 (59.0–85.5)	72.0 (56.5–82.0)	0.009 *
NT-proBNP, pg/mL ***	5194 (988–5194)	833 (422–2023)	893 (434–2215)	0.062
Hemoglobin, mmol/L ***	6.7 (5.1–7.4)	6.8 (6.0–7.6)	6.8 (6.0–7.5)	0.74
**Outcome**				
Postoperative pacemaker implantation	0 (0%)	21 (15.6%)	21 (14.9%)	0.59
Postoperative stroke	1 (16.7%)	3 (2.2%)	4 (2.8%)	0.16
Bleeding requiring transfusion (*n* = 139)	1 (16.7%)	9 (6.8%)	10 (7.2%)	0.37
Acute kidney injury (*n* = 95)	2 (40%)	16 (17.8%)	18 (12,8%)	0.24
Hemodynamic relevant pericardial effusion	1 (16.7%)	0 (0%)	1 (0.7%)	0.04 *
Died during procedure	0 (0%)	0 (0%)	0 (0%)	>0.99
ICU admission (*n* = 134)	1 (16.7%)	6 (4.7%)	7 (5.2%)	0.28
Died in hospital	0 (0%)	1 0(.7)	1 (0.7%)	>0.99
Length of stay in hospital, days	15.0 (13/26)	10 (8/13)	10 (8/13)	0.003 *
Discharge home	3 (50%)	122 (90.4%)	125 (88,7%)	0.02 *
Discharge rehabilitation	2 (33.3%)	3 (2.2%)	5 (3,5%)	0.01 *
Discharge to nursing home	0 (0%)	1 (0.7%)	1 (0,7%)	>0.99
Discharge to another hospital	1 (16.7%)	8 (5.9%)	9 (6,4%)	0.33
**Follow-Up**				
Subjective feeling of no change in cognitive function	1/1 (100%)	28/114 (24.6%)	87/116 (82.3%)	0.44
Died within 3 months	0/5 (0%)	3/128 (2.3%)	3/133 (2.3%)	>0.99
Rehospitalization within 3 months	2/3 (66.7%)	21/100 (21%)	23/103 (22.3%)	0.12
Died within 6 months	1/6 (16.7%)	4/88 (4.5%)	5/94 (5.4%)	0.01

PTCA, percutaneous transluminal coronary angioplasty; RVP, rapid ventricular pacing; TAPSE, tricuspid annular plane systolic excursion; LVEF, left ventricle ejection fraction; CABG, coronary arterial bypass grafting. Self-expanding valve: Evolut™, ALLEGRA™, ACURATE neo™, Portico™. Balloon-expanding valve: SAPIEN™. Died within 3 months missing values: 8/141; died within 6 months missing values: 49/141; * significant *p*-value; ** 24–48 h after TAVR; *** laboratory markers at discharge.

**Table 3:** Memory Function by POD.

	With POD	Without POD	Overall	*p*-Value
**Preoperative memory function**				
WL learning	*n* = 6	*n* = 135	*n* = 141	
Number of recalled words	13 ± 4.0	14 ± 3.8	14 ± 3.8	0.48
z-value	−2.3 ± 1.6	−1.4 ± 1.1	−1.45 ± 1.1	0.08
WL recall	*n* = 6	*n* = 134	*n* = 140	
Number of recalled words	2 ± 2.2	2 ± 2.2	2 ± 2.2	0.88
z-value	−2.8 ± 1.3	−2.3 ± 1.1	−2.4 ± 1.1	0.48
DST	*n* = 6	*n* = 135	*n* = 141	
Digit span	5 ± 0.8	5 ± 1.0	5 ± 1.0	0.11
**Pre-/postoperative difference scores (t1–t0)**				
WL learning	*n* = 3	*n* = 109	*n* = 112	
Difference score number of recalled words	0 ± 5.0	2 ± 3.2	2 ± 3.2	0.66
WL recall	*n* = 3	*n* = 108	*n* = 111	
Difference score number of recalled words	0 ± 0.6	0 ± 1.9	0 ± 1.9	0.25
DST	*n* = 3	*n* = 110	*n* = 113	
Difference score digit span	0 ± 0.6	0 ± 0.9	0 ± 0.9	0.40

WL: word-list task (CERAD), DST: digit span task.

## Data Availability

Data available on request due to restrictions, e.g., privacy or ethical reasons. The data presented in this study are available on request from the corresponding author. The data are not publicly available due to privacy restrictions.
